# Supplementary material for: Irinotecan (CPT-11) Canonical Anti-Cancer Drug Can also Modulate Antiviral and Pro-Inflammatory Responses of Primary Human Synovial Fibroblasts
Source: Cells. 2021 Jun 8;10(6):1431. doi: 10.3390/cells10061431 (PMC8230279; doi:10.3390/cells10061431)
Supplement: Supplementary file 1 [file cells-10-01431-s001.zip › Added Supplementary_Materials_Dobi_et_al_FigureS2.pdf]

SUPPLEMENTARY MATERIALS

A)

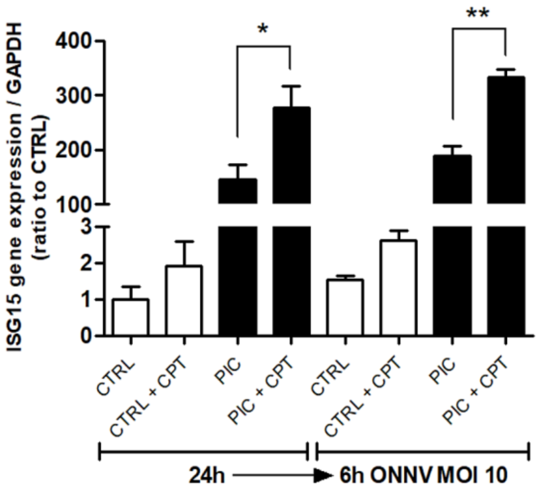

B)

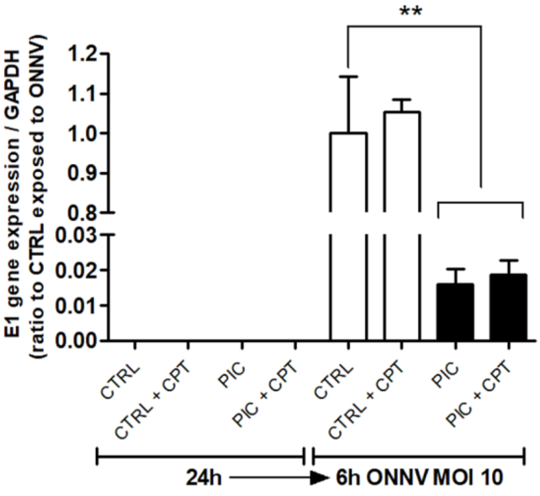

C)

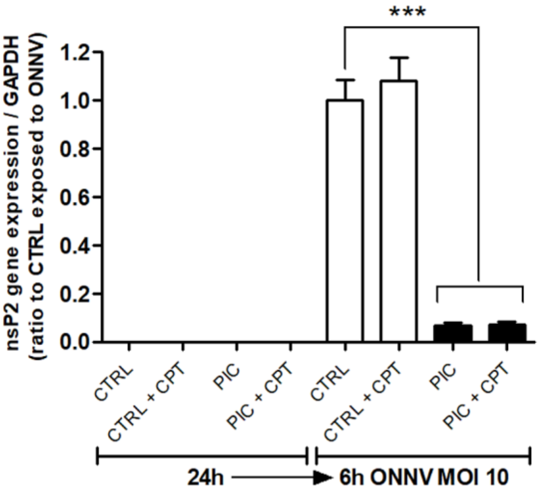

Figure S2. CPT-11 does not interfere with the blunted ONNV replication observed in HSF previously exposed to PIC. A) ISG15, **B)** E1 and **C)** nsP2 mRNA levels from HSF treated with PIC (1 µg/mL) for 24 hours, in the presence or absence of CPT-11 (15 µM), and then exposed for 6 hours to ONNV (MOI 10), were evaluated by RT-qPCR. Results are expressed as mean ± SEM of three independent experiments. Statistical significance is indicated as follows: p-value < 0.05 (*), p-value < 0.01 (**), p-value < 0.001 (***).
