# Supplementary material for: Irinotecan (CPT-11) Canonical Anti-Cancer Drug Can also Modulate Antiviral and Pro-Inflammatory Responses of Primary Human Synovial Fibroblasts
Source: Cells. 2021 Jun 8;10(6):1431. doi: 10.3390/cells10061431 (PMC8230279; doi:10.3390/cells10061431)
Supplement: Supplementary file 1 [file cells-10-01431-s001.zip › Revised Supplementary_Materials_Dobi_et_al_FigureS1.pdf]

SUPPLEMENTARY MATERIALS

A)

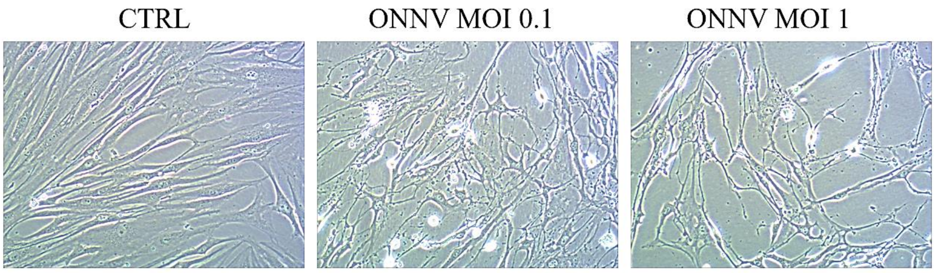

B)

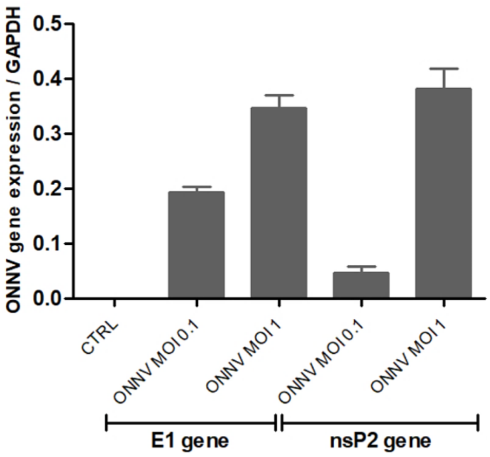

C)

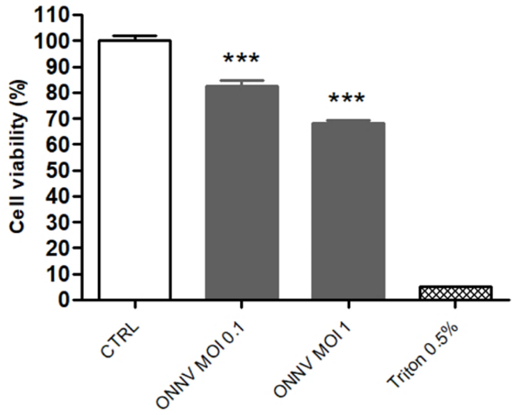

D)

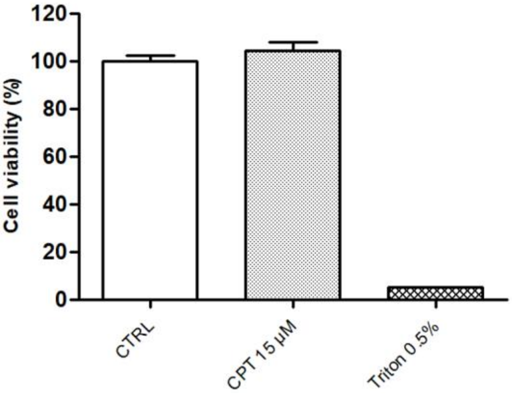

E)

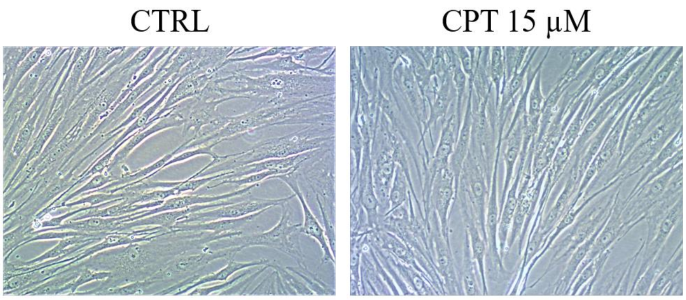

Figure S1. ONNV can replicate in HSF and alters cell viability. CPT-11 at 15 µM does not affect cell viability. A) HSF morphology was observed by phase-contrast microscopy (magnification x100). **B)** E1 and nsP2 mRNA levels from HSF treated with ONNV (at MOI 0.1 and 1), for 24 hours, were evaluated by RT-qPCR. **C-D)** Cell viability was measured using the MTT colorimetric assay 24 hours after cell treatment. **E)** HSF morphology was observed after 24 hours treatment with CPT-11. Quantitative results are expressed as mean ± SEM of four independent experiments. Statistical significance is indicated compared to control (CTRL), as follows: *p*-value < 0.001 (***).
