# Supplementary material for: Irinotecan (CPT-11) Canonical Anti-Cancer Drug Can also Modulate Antiviral and Pro-Inflammatory Responses of Primary Human Synovial Fibroblasts
Source: Cells. 2021 Jun 8;10(6):1431. doi: 10.3390/cells10061431 (PMC8230279; doi:10.3390/cells10061431)
Supplement: Supplementary file 1 [file cells-10-01431-s001.zip › Supplementary_Materials_Dobi_et_al_FigureS3.pdf]

SUPPLEMENTARY MATERIALS

A)

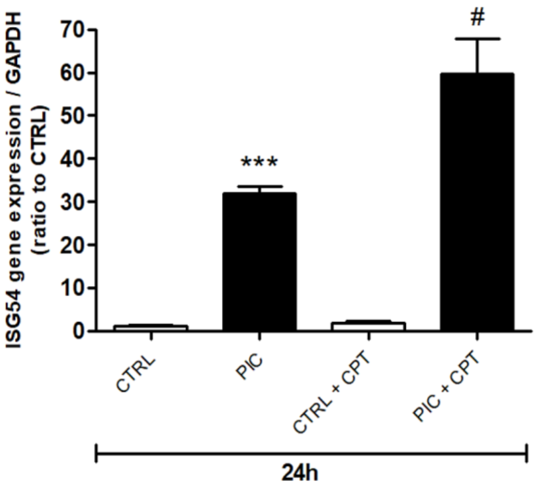

B)

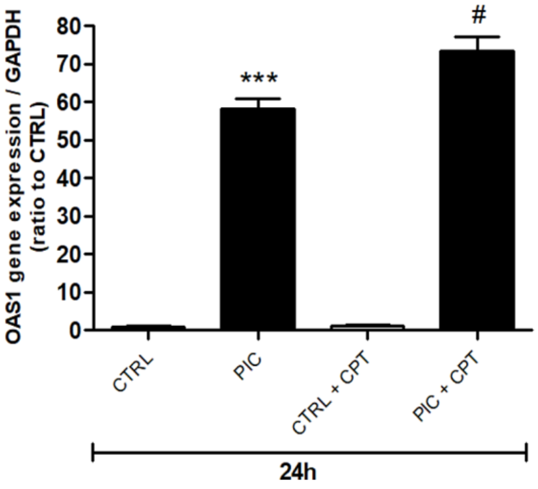

C)

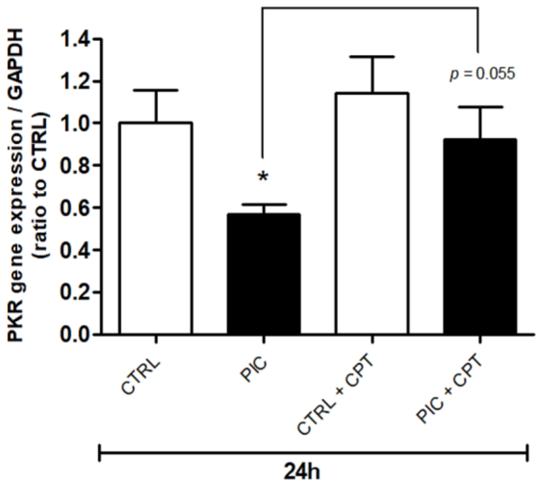

Figure S3. CPT-11 enhances several ISGs expression in PIC-stimulated HSF. A) ISG54, **B)** OAS1 and **C)** PKR mRNA levels from HSF co-treated with PIC (1 µg/mL) and CPT-11 (15 µM), for 24 hours, were evaluated by RT-qPCR. Results are expressed as mean ± SEM of four independent experiments. Statistical significance is indicated as follows: *p*-value < 0.05 (*), *p*-value < 0.001 (***) compared to control at the corresponding time of treatment; *p*-value < 0.05 (#) compared to the corresponding treatment without CPT-11.
