# Supplementary material for: Irinotecan (CPT-11) Canonical Anti-Cancer Drug Can also Modulate Antiviral and Pro-Inflammatory Responses of Primary Human Synovial Fibroblasts
Source: Cells. 2021 Jun 8;10(6):1431. doi: 10.3390/cells10061431 (PMC8230279; doi:10.3390/cells10061431)
Supplement: Supplementary file 1 [file cells-10-01431-s001.zip › Supplementary_Materials_Dobi_et_al_FigureS4.pdf]

SUPPLEMENTARY MATERIALS

A) **B)**

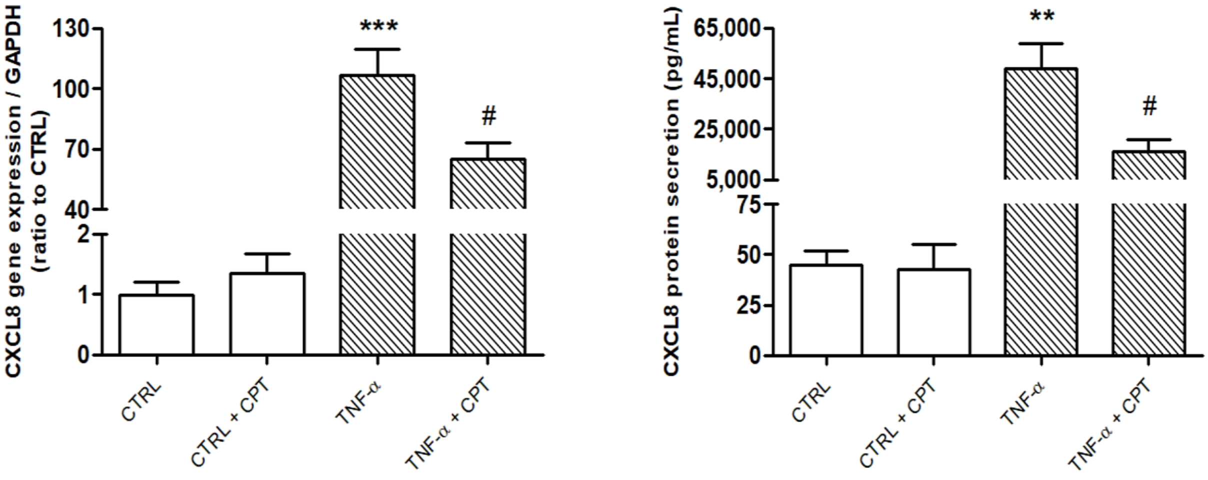

Figure S4. CPT-11 reduces TNF-α-induced CXCL8 expression. A) CXCL8 mRNA and **B)** protein levels from HSF co-treated with TNF-α (50 ng/mL) and CPT-11 (15 µM), for 6 hours, were evaluated by RT-qPCR and ELISA respectively. Results are expressed as mean ± SEM of three independent experiments. Statistical significance is indicated as follows: *p*-value < 0.01 (**), *p*-value < 0.001 (***) compared to control at the corresponding time of treatment; *p*-value < 0.05 (#) compared to the corresponding treatment without CPT-11.
